# The Specialist before a General Medical Society

**Published:** 1893-05

**Authors:** 


### EDITORIAL.

THE following quotation from the constitution of the American Medical Association defines very clearly the status of the properly educated dentist in respect to the American Medical Association. This part of the constitution was introduced some eight or nine years ago, by Dr. N. S. Davis, of Chicago, at the meeting of the Association, at Richmond, Va. The amendment was passed at the next meeting of the Association, held in Chicago; it was advocated by Drs. Davis, of Chicago, Lewis Sayre, of New York, and a number of other leading physicians of the country, and when brought to a vote was passed unanimously. This not only fixes the status of the dentist in respect to the American Medical Association, but practically does so for all other State or local associations. There would be no propriety in any State or local society refusing to receive to membership any one who was eligible to membership in the American Medical Association. The course of study and work required in all our best colleges fully meets this requirement. The American Medical Association has its section on Oral and Dental Surgery, which occupies exactly the same position as any other section of that body.

We ask special attention to the amendment which is as follows:

"Members of Canadian and Mexican medical societies shall be admitted to membership upon the same terms as those in the United States. The regular graduates of such schools and colleges of dentistry as require of their students, a standard of general education and a term of professional study equal to the best class of medical colleges in this country, and embrace in their curriculum all the fundamental branches of medicine; differing chiefly by substituting practical and clinical instruction in oral and dental surgery in place of practical and clinical instruction in general medicine and surgery, are recognized as members of the regular profession and eligible to membership upon the same terms as other members."

### The Specialist Before a General Medical Society.

"When a specialist appears before a society composed mainly of practitioners of medicine and surgery, it should be with one of three avowed intentions, either first, to present new investigations or discoveries which can not be better presented elsewhere; or second, to instruct the members in that which it is important for them to know; or third, simply to remind his hearers that he is still "doing business at the old stand." The numerous local and national special associations and special sections of general associations afford more than sufficient space for the presentation of all discoveries and investigations in special fields of labor, and in such meetings only can they be properly appreciated and intelligently discussed; and the specialist who takes the results of such work into a meeting of general practitioners subjects himself, unjustly no doubt in most instances, to the criticism of covert advertising. That any physician will selfishly occupy the time of any meeting solely for the sake of gain we cannot believe, although the regularity with which some reappear at the surface would seem to justify this conclusion. The only excuse, then, for the specialist would seem to be that he comes in the guise of a teacher and is prepared to impart knowledge which the members wish or need. As a general thing the society, or its officers, are competent to judge of these needs and wishes."

The above is copied from an editorial in the *Journal of the American Medical Association*, of April 1st., and seems to define very definitely what a specialist ought to do if he ventures into a general medical society. It says he is there first, "to present new investigations or discoveries which cannot be better presented elsewhere," or second, "to instruct the members in that which it is important for them to know, or third, simply to remind his hearers that he is still doing business at the old stand." Now why does any one, special or general practitioner, go to a medical society? Is it not to be helped and to help others? The true specialist is educated in the fundamental principles of medical science, and he is interested in these, and in the progress made in them just as much as the general practitioner.

The specialist may not be able to present any new investigations or discoveries, he may not be able to instruct the members in that which it is important for them to know, but he himself may gain very much from that which others have to give, and what reason shall we give for excluding him from such an opportunity? The same reason for excluding him would, in many instances, exclude the general practitioner. If only general practitioners are admitted to medical societies who have some new investigations or discoveries to present, or are able "to instruct the members in that which it is important for them to know," the membership of many medical societies would be very much reduced indeed.

Will not the facts sustain the statement that specialists in medical societies oftentimes have matters to present of interest and of practical utility to the general practitioner? The truth is, every specialist needs to know as much as possible of the entire range of medical science and of the progress that is being made, and the general practitioner will, at least, not be injured by knowing much that the specialist may impart to him.

The third item here "simply to remind his hearers, etc." really does not belong here, it is almost a sneer at the specialist. Is the specialist more likely "simply to remind, etc." than the general practitioner? It is true that many specialists seek to advertise themselves in all practical ways; are there not, however, some general practitioners who seize all opportunities to have it known that they are still "doing business at the old stand?"

We would suggest (a mere suggestion) that brother Culbertson revise this editorial.

